# Water soluble organic aerosols in the Colorado Rocky Mountains, USA: composition, sources and optical properties

**DOI:** 10.1038/srep39339

**Published:** 2016-12-19

**Authors:** Mingjie Xie, Natalie Mladenov, Mark W. Williams, Jason C. Neff, Joseph Wasswa, Michael P. Hannigan

**Affiliations:** 1 Department of Mechanical Engineering, University of Colorado, Boulder, CO 80309, United States; 2 Department of Civil, Construction, and Environmental Engineering, San Diego State University, San Diego, California 92182, United States; 3 Institute for Arctic and Alpine Research and Department of Geography, University of Colorado, Boulder, CO 80309, United States; 4 Department of Geology, University of Colorado, Boulder, CO 80309, United States

## Abstract

Atmospheric aerosols have been shown to be an important input of organic carbon and nutrients to alpine watersheds and influence biogeochemical processes in these remote settings. For many remote, high elevation watersheds, direct evidence of the sources of water soluble organic aerosols and their chemical and optical characteristics is lacking. Here, we show that the concentration of water soluble organic carbon (WSOC) in the total suspended particulate (TSP) load at a high elevation site in the Colorado Rocky Mountains was strongly correlated with UV absorbance at 254 nm (Abs254, *r* = 0.88 *p* < 0.01) and organic carbon (OC, *r* = 0.95 *p* < 0.01), accounting for >90% of OC on average. According to source apportionment analysis, biomass burning had the highest contribution (50.3%) to average WSOC concentration; SOA formation and motor vehicle emissions dominated the contribution to WSOC in the summer. The source apportionment and backward trajectory analysis results supported the notion that both wildfire and Colorado Front Range pollution sources contribute to the summertime OC peaks observed in wet deposition at high elevation sites in the Colorado Rocky Mountains. These findings have important implications for water quality in remote, high-elevation, mountain catchments considered to be our pristine reference sites.

Most particulate matter (PM) monitoring focuses on smaller particle sizes, such as PM < 2.5 μm in diameter (PM_2.5_) and PM < 10 μm (PM_10_) in diameter, which are important for human health. The vast majority of PM, however, is found in larger size classes that can be captured in the total suspended particulate (TSP) load and includes constituents such as dust, biological organisms, organic and inorganic debris, and other primary and secondary aerosols, which may be relevant for aquatic and soil ecosystem biogeochemical processes.

For remote alpine settings, where terrestrial carbon (C) and nutrient inputs from the catchment are less prominent than in forested catchments^1^, for example, studies have shown that PM deposition of dust and organic material can be an important source of calcium^2^,^3^, phosphorus^4^, organic carbon^5^ and bioaerosols^6^. However, atmospheric aerosols can also be a source of air pollutants to alpine environments^7^,^8^,^9^, which can have deleterious effects on ecosystem health and water quality in sensitive alpine ecosystems. Inputs of organic carbon, in particular, can influence redox reactions in the water column and sediments and can fuel microbial activity resulting in unanticipated consequences for otherwise isolated waters that we regard as our pristine reference sites. For the Rocky Mountains, studies have shown that the organic carbon load in atmospheric deposition is substantial^1^,^10^, but direct evidence of the sources of organic aerosols is thus far lacking.

Organic PM generally constitutes a substantial fraction (20–50%) of atmospheric aerosols^11^ and could be classified into water insoluble organic carbon (WIOC) and water soluble organic carbon (WSOC)^12^. Studies have shown that WIOC has a similar diurnal profile to that of elemental carbon (EC)^13^,^14^, suggesting an origin of primary emissions, including biomass and fossil fuel combustion. By contrast, WSOC has both primary (e.g., biomass burning) and secondary (e.g., secondary organic aerosol formation) sources^15^,^16^, and can account for a substantial fraction (20–80%) of particulate organic matter on a carbon mass basis^17^,^18^. Water soluble components like WSOC may support biogeochmical processes by serving as an energy source for soil microbial communities. In the atmosphere, these water soluble constituents have an important influence on the hygroscopicity of atmospheric aerosols^19^. The hygroscopic behaviors of particles play an important role in aerosol radiative properties and lifetime as well as cloud nucleating properties^20^,^21^.

The WSOC pool is a complex mixture of compounds likely containing oxygenated functional groups such as carboxyl, hydroxyl and carbonyl groups^17^. Based on compound-specific techniques such as gas chromatography coupled with mass spectrometry (GC-MS), several classes of chemical compounds have been identified (e.g., saccharides and dicarboxylic acids) and used as tracers for specific emission sources^22^,^23^,^24^. Spectroscopic techniques such as ultraviolet (UV)-visible absorbance spectroscopy and excitation-emission matrix (EEM) fluorescence spectroscopy has been widely used to investigate the fluorescent properties of dissolved organic matter (DOM) in aquatic environments^5^,^6^,^25^ but less so for WSOC in aerosols^26^. Fu *et al*.^27^ observed temporal variations between fluorescence peaks and primary organic tracers (e.g., PAH, levoglucosan and sucrose) in ambient aerosols collected from the Arctic region, indicating sources of biomass burning, fossil fuel combustion and primary biological aerosols for fluorescent organic aerosols. Parallel factor analysis (PARAFAC), a powerful multi-way modeling tool, is used to resolve EEMs into several individual fluorescent groups, which could reflect the origins (e.g., fulvic-like, humic-like) of chromophoric DOM (CDOM) in aquatic ecosystems^28^,^29^. Using PARAFAC modeling, Mladenov *et al*.^26^ found three dominant fluorescent components, including one that was representative of diesel fuel-derived WSOC in PM_10_ samples from an urban environment.

Apportioning the sources of WSOC is relevant for improving our understanding of the chemical quality and seasonality of aerosol deposition as well as preparing for future environmental and climatic changes that may influence organic aerosol loadings to alpine environments. Regularly occurring peaks in DOC concentrations in wet deposition, as high as 10 mg C L^−1^ (Mladenov *et al*.^1^), have been observed in summertime wet deposition at several collection sites (CO02, CO90, and CO94, Supplementary Fig. S1) operated by the US National Atmospheric Deposition Program (NADP) and at the Niwot Ridge Long Term Ecological Research (NWT-LTER) station (Soddie site, Fig. 1) in the Colorado Rocky Mountains. Based on significant correlations between DOC concentrations and sulfate and nitrate in summer wet deposition^1^, air mass trajectories that pass through the Front Range of Colorado in the summer^10^, and summer upslope conditions that carry air masses above the atmospheric boundary layer, the authors hypothesized that the summertime organic aerosol load in high elevation site in the Colorado Rocky Mountains is substantially contributed by Colorado Front Range derived anthropogenic primary and secondary organic aerosol (SOA) emissions.

To test this hypothesis and to identify seasonal variability in the sources of organic aerosols, we analyzed the chemical characteristics of organics in aerosols collected by a TSP collector instrumented near tree line at the Sodde site (Fig. 1). Over a ~two-year period, we measured total organic carbon (OC), elemental carbon (EC) and an array of water soluble organic molecular markers (WS-OMMs). On the water soluble fraction extracted from the TSP filters, we measured WSOC, water soluble nitrogen (WSN) and water soluble phosphorus (WSP) concentrations; UV-vis absorbance and fluorescence spectroscopic characteristics of the water extracts were also obtained. To further identify and quantify potential sources contributing to the organic aerosols, we also conducted positive matrix factorization (PMF) of selected chemical speciation data, which allowed us to partition specific input sources of WSOC at this high elevation site.

## Results

### Bulk components

Measurements of bulk species concentrations, including WSOC, OC, EC, WSN, WSP and TSP, at the high elevation site (Table 1) indicate that >90% of the OC in TSP samples was water soluble. WSOC and OC had median concentrations of 348 ng m^−3^ and 373 ng m^−3^, respectively, although concentrations >1000 ng m^−3^ were measured for both. Concentrations of EC, which typically represents fossil fuel combustion^30^, were much lower, ranging from 0.0023 to 439 ng m^−3^ (median of 23.3 ng m^−3^). The mean concentration of WSN (88.8 ng m^−3^) was 1–2 orders of magnitude higher than the mean WSP concentration (1.18 ng m^−3^). Temporal variations of the above bulk components (Fig. 2a–f) indicate that a major increase in concentrations occurs during the summer period for all bulk components. A pronounced spike was observed for EC and WSOC concentrations, corresponding to the sample collected from 31 May–7 June 2011. Concentrations of the other 4 components (OC, WSN, WSP and TSP) also had an increase during that period.

### WS-OMMs

Based on GC-MS technique, A series of WS-OMMs in the TSP samples were quantified using GC MS techniques. Three homologous dicarboxylic acids (C4–C6), detected in the high elevation aerosols (Table 1), reflect secondary photochemical reactions and/or primary emissions of fossil fuel combustions^22^,^31^. Succinic acid had the highest concentration (median of 3.32 ng m^−3^), followed by glutaric acid (1.10 ng m^−3^) and adipic acid (0.65 ng m^−3^). Three hydroxy dicarboxylic acids, also used as source markers for SOAs^32^,^33^, were detected in our samples, including malic acid, hydroxyglutaric acid and hydroxyadipic acid, with median concentrations of 0.91, 0.82, and 0.44 ng m^−3^, respectively.

Three compounds previously used as isoprene SOA tracers, 2-methylglyceric acid, 2-methylthreitol and 2-methyletythritol^34^,^35^, were detected in our samples, at concentrations ranging from 0.0061 to 14.3 ng m^−3^. Levoglucosan, mannosan and galactosan have been widely used as organic tracers for biomass burning^36^,^37^. These compounds were detected in all collected samples, at concentrations ranging 0.018–10.9 ng m^−3^. Three sugars (mannose, fructose and glucose), two sugar alcohols (meso-erythritol and arabitol) and three sugar acids (glyceric acid, erythronic and threonic acids) were also detected. Their median concentrations ranged from 0.026 to 0.73 ng m^−3^, comparable to other polar organic tracers.

Concentrations of all polar organic tracers except anhydro sugars, which are associated with biomass burning emissions, increased rapidly after May 2011 and remained high for most of the summer until October 2011 (Fig. 2g–l,). Consistent with the spike observed for EC and WSOC, we also observed a sharp increase for andydro sugar (16.4 ng m^−3^) during the 31 May–7 June 2011 period. This spike was not observed for other polar organic tracers. Excluding the spike, anhydo sugar did not show an obvious seasonal trend.

### UV-light absorbing and fluorescent properties

The optical properties, UV-visible absorbance and fluorescence, listed in Table 1 for WSOC samples, provide additional information about light absorbance and presence of aromatic and double-bonded C structures. The light absorption coefficient at 254 nm (Abs254) ranged from 0.24 to 6.36 Mm^−1^ with a median value of 0.69 Mm^−1^; the bulk mass absorption coefficient at 254 nm (MAC254) has a value ranging from 0.73 to 5.58 m^2^ g^−1^C with a median of 2.30 m^2^ g^−1^ C. The florescence index (FI) ranged from 1.18 to 1.57 with a median value of 1.40. The ranges of humification index (HIX) and freshness index (*β:α*) are 0.72–4.75 (median 2.32) and 0.54–0.75 (0.65), respectively.

The spike observed during 31 May–7 June 2011 for WSOC, EC and anhydro sugar concentrations was also reflected in the high Abs254 and MAC254 values during the same period (Fig. 3a,b). The Abs254 value also exhibited an overall increase during summer periods. FI has no seasonal trend and only minor variations (Fig. 3c). Similar to the results for FI, values of HIX and *β:α* did not have seasonal trends, but their variations were larger than for FI (Fig. 3d,e). These two parameters were negatively correlated (*r* = −0.62, *p* < 0.01).

### PMF results

A 3-factor solution (SOA formation, biomass burning and motor vehicle emission) was selected due to its most interpretable resulting factors and high factor matching rate (83.8%) between bootstrapped and base case solutions. If we were to select a factor number bigger than 3, then the factor matching rate would have decreased and some of the replicate data sets could not have converged PMF solutions. Figures 4 and 5 exhibit the factor profiles and factor contributions to WSOC for the 3-factor solution. The factor profile shown in Fig. 4 has been normalized by


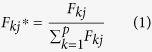


where F^*^_kj_ is the relative weighting of specie *j* in factor *k* to other factors. The overall factor contributions to WSOC agreed with the observed WSOC concentrations (PMF simulation/observation = 0.95 ± 0.20, *r* = 0.93, *p* < 0.01).

PMF results indicate that biomass burning factor had higher average contributions to WSOC (50.3%) than the other two factors, SOA formation (21.9%) and motor vehicle emissions (27.8%). However, the SOA formation factor dominated the contribution to WSOC in summer periods (Fig. 5). The SOA formation factor contains the highest percentages of dicarboxylic acid, hydroxy dicarboxylic acid, isoprene SOA and sugar acid (Fig. 4). The biomass burning factor was characterized by the highest percentages of anhydro sugar, and EC was almost uniquely loaded in the motor vehicle emission factor (Fig. 4).

## Discussion

The WSOC concentrations exhibited maxima in the summer and minima in winter (Fig. 2a), which is consistent with seasonal trends (summer maxima, winter minima) for DOC in wet deposition observed by Mladenov *et al*.^1^ at the same sampling site and at other NADP sampling stations in the vicinity (Supplementary Fig. S1). The average concentration of WSOC (406 ng m^−3^) observed in this study is lower than that observed at Tibetan Plateau (770 ng m^−3^)^38^, but higher than that in high Arctic atmosphere (186 ng m^−3^)^27^. Without considering the wildfire-impacted sample, WSOC accounted for a substantial part (mean ± std, 91.1 ± 19.5%) of OC, and was strongly correlated with OC (*r* = 0.95, *p* < 0.01) and amount of light absorbing compounds, reflected in the Abs254 parameter (*r* = 0.88, *p* < 0.01). Given the high summer factor contribution of the SOA formation factor described by the PMF analysis (Fig. 5), the summer time peaks of WSOC and OC may be caused by formation of SOA through atmospheric reactions.

In this study, multiple peaks of EC concentrations were observed in summer periods (Fig. 2c). Without the influence of biomass burning, EC observed in high mountain areas would be expected to come from urban-derived vehicle emissions through short- or long-term transport^39^,^40^. The increase in temperature during summer leads to a decrease in air density, which causes a substantial increase in PM released from heavy-duty diesel engines^41^. For instance, Chen *et al*.^42^ observed summer peaks in EC source strength at Fort Meade, Maryland, and found that the EC emission increased with temperature once the temperature exceeded a threshold. For our study, the Denver metropolitan area is about 80 km to the southeast of the sampling site, and the air masses more frequently originate from the south and southwest in summer periods, as shown in the backward trajectory analysis results (Supplementary Figs S2 and S3). In addition, summer upslope wind conditions are known to prevail in the summer, and these conditions may transport Front Range-derived EC, shown to have increased source strength in the summer^43^, to higher elevations. As such, emissions from the urban area in and around Denver may contribute to the multiple peaks of EC concentrations observed at our site in the summer and may, therefore, also play a role in summertime WSOC and OC inputs.

Our results also reflect a notable influence of secondary organic aerosol formation on WSOC. Low molecular weight dicarboxylic acids in the atmosphere have both primary and secondary sources. The concentrations of the three dicarboxylic acids in this work were 1–2 orders of magnitudes lower than those observed in urban areas^44^,^45^,^46^. In Fig. 5, dicarboxylic acid has the highest loading on the SOA formation factor, indicating the dominance of secondary formation rather than primary emissions; however, we cannot rule out its formation from anthropogenic precursors^47^. Hydroxy dicarboxylic acids, previously demonstrated as source markers for monoterpene and cyclohexene derived SOA^32^,^33^, and isoprene SOA compounds, also exhibited summer time maxima (Fig. 2). Air mass backward trajectory analysis, conducted for every other day from July 05, 2011 to August 02, 2011 to verify the general source region of air masses, showed south (urban Denver) and southwest Colorado (mostly covered by forest) as the most dominant source area (Supplementary Fig. S3). So both biogenic and anthropogenic emissions may contribute to the SOA formation at the sampling site.

Soil and/or dust re-suspension is an important source for TSP^48^,^49^, and the organic carbon associated with dust deposition has been demonstrated as an input for DOM in alpine lakes^6^,^50^. The enhancement of WSN in summer periods might be related to the formation of water soluble inorganic nitrogen (WSIN, e.g., NO_3_^−^) by the reaction of mineral aerosols with gaseous nitric acid^51^,^52^. Water soluble organic nitrogen (WSON, e.g., nitrocatechol) could be formed through the photo-oxidation of aromatic VOCs (e.g., toluene, m-cresol) under the presence of NO_X_^53^,^54^. Also, soil and/or dust re-suspension are known sources of P in deposition^55^ and may contribute to the summertime increase in WSP we observed. The contribution of soil and/or dust re-suspension was also suggested by the higher concentrations of sugars and sugar alcohols in summer than in other periods. These compounds are associated with the activity of microorganisms in soil and dust^23^,^56^. Similar seasonal trends of total dissolved nitrogen and dissolved phosphorus in wet deposition have been observed by Mladenov *et al*.^1^ and Oldani^10^ at the same sampling site. The sample collected during 16–30 November 2010 has the highest concentration of TSP (Supplementary Table S1) and much lower mean and median OC and WSOC concentrations than the other samples, suggesting a contribution from dust enriched with inorganics.

Wildfire had a measureable influence on TSP characteristics in this study. The sample collected during 31 May–7 June 2011 was observed to have spikes in the concentrations of WSOC, EC, and anhydro sugars and in the Abs254 and MAC254 values (Figs 2a,c,j and 3a, b). The three anhydro sugars detected in this work are mainly produced during the pyrolysis of cellulose/hemicelluloses^36^,^37^ and are commonly used tracers for biomass burning^57^. Biomass burning can also contribute to WSOC and EC in ambient aerosols^58^, which will impact the hygroscopicity and radiative forcing of atmospheric aerosols. The light absorbance at 254 nm reflects aromatic moieties^59^ as does MAC254, the normalized value of A254 to the DOC concentration^60^. The MAC254 value of this sample (5.58 m^2^ g^−1^C) had the highest value of all samples, and was more than twice as high as the median (2.30 m^2^ g^−1^C) and average (2.52 m^2^ g^−1^C). The strong light absorbance at 254 nm could be related to the high WSOC concentration with lignaceous and other aromatic compounds from biomass burning.

Fluorescence properties also reflect the influence of biomass burning aerosols during the June 2011 period. The June 7, 2011 sample from this period had the highest HIX ratio (at 4.75) and third lowest *β:α* ratio (at 0.57), which are indicative of high C:H molar ratios and more aged organic material, respectively. The EEM of this sample also resembled EEMs of burned samples of upland soils that had been leached in ultrapure water and UV irradiated for 30 d^61^. In that study, irradiation had photobleached most of the peak C fluorescence, leaving behind organic material with fluorescence only present in a region centered at 250 nm excitation and 430 nm emission. Our PARAFAC components C1 and C2 occupy that similar humic-like fluorescence region (centered at ~250 nm excitation and 412 nm and 486 nm emission, respectively) and represented 66% of the total fluorescence in the 7 June 2011 sample (Supplementary Table S2).

We investigated the possible sources of biomass burning aerosols for the region during this period. The Wallow fire in Arizona in early June 2011 was a major source of smoke to much of the US, and NASA MODIS imagery shows plumes passing directly over the study site in Colorado (Supplementary Fig. S4). Air masses passing through Arizona and the Four Corners Region, USA (as shown in backward trajectories, Supplementary Fig. S5) may have carried biomass burning aerosols to the Niwot Ridge study site. As such, the sample collected during 31 May–7 June 2011 likely reflects the impact of wildfire smoke from Arizona.

Based on the PMF analysis, biomass burning provided the greatest contributions to WSOC on an annual basis. In summer periods, SOA formation dominated the contribution to WSOC, and motor vehicle emission also had significant contributions to WSOC intermittently in warm periods. Given the limited number of source markers and samples available for source apportionment in our study, it is possible that some important source-related factors may have been combined into one, missed, or misclassified. For example, the SOA formation factor lumps both biogenic and anthropogenic SOA contributions together. Also, soil/dust associated organics can contribute to WSOC in atmospheric aerosols and usually have enhanced contributions during the spring and summer^23^,^62^. Missing this factor/source could lead to an overestimation of contributions for the three resolved factors in Fig. 5. Nevertheless, the three resolved factors do represent the most important sources of WSOC in ambient aerosols^63^. In addition, the temporal variations of the three factor contributions are not expected to change should additional factors/sources be resolved.

Our results further indicate that there are challenges with the application of fluorescence spectroscopy to the study of organic aerosols extensively modified by photochemical processes. One of the most noticeable trends in EEMs of our samples is the absence of fluorescence at higher excitation wavelengths (>350 nm), which is evident in both the EEM plots of PARAFAC modeled components (Supplementary Fig. S6) and representative samples (Supplementary Fig. S7). The removal of fluorescent WSOC that absorbs light at higher wavelengths has been shown to result from photochemical processes acting to transform organic compounds^64^, and this would directly affect the FI, which is calculated at an excitation of 370 nm. Also, FI values decrease as a result of photobleaching of OM^64^. Therefore, the low FI values in our study are more likely to be explained by photobleaching than by the presence of terrestrially-derived DOM, which is typically associated with low FI values^65^.

In addition, we also did not see significant correlations between any of the four PARAFAC fluorescent components (Supplementary Table S2) and any of the three PMF factors or major TSP components. Given that optically active compounds in organic matter are sensitive to photochemical processes, such as photobleaching, the lack of correlations between fluorescence and WSOC sources is not unexpected. Irradiation induces loss of fluorescence that is highest at the irradiation wavelength^66^, which occurs at higher wavelengths for larger structures, such as humic and fulvic acids^67^. Therefore, we advise caution when using fluorescence spectroscopy to characterize atmospheric aerosols, especially those that may undergo substantial phototransformation due to long transport times in the atmosphere. It remains to be seen whether the effects of photolysis on WSOC fluorescence is less pronounced for samples collected in close proximity to their aerosol source (e.g., urban air pollution or regional dust transport). Relationships between absorbance at the lower wavelength of 254 nm and other WSOC properties were not notably affected.

Indeed, most of the correlations between Abs254 and chemical components were significant (Supplementary Fig. S8). Without considering the wildfire impacted sample, which has very high Abs254 likely due to the presence of aromatic C generated during fire^68^, Abs254 is strongly correlated with WSOC (*r* = 0.88, *p* < 0.01) and SOA markers (*r* > 0.70, *p* < 0.01). If the wildfire impacted sample had been included, the correlations of Abs254 versus WSOC and SOA markers became weaker and even insignificant, respectively; the slopes of regressions became much smaller. These results suggest that the organic compounds emitted from biomass burning had much stronger UV absorbance than those from SOA formation.

This study leveraged multiple analytical and statistical techniques to investigate the chemical composition, optical properties and sources of organic carbon in TSP samples collected at a high elevation site in the Rocky Mountains. Our results indicate that biomass burning, SOA formation (with both biogenic and anthropogenic SOA), and motor vehicle emission are the main sources contributing to WSOC. Our analyses could not separate out the dust related sources, which we expect to be more prominent in the warmer spring and summer months. Nevertheless, the important contribution of SOA formation, especially to summertime WSOC in TSP samples, and the multiple summertime peak contributions of anthropogenic emissions (as EC) supported that anthropogenic (e.g., Colorado Front Range) sources of pollution contribute substantially to the previously reported high summertime DOC in wet deposition at NWT-LTER. Additionally, given that increasing temperatures are expected to increase monoterpene SOA generation and exacerbate wildfire activity in the Western USA, thereby increasing summertime OC and EC aerosol concentrations^69^, we can expect even higher future loadings of carbonaceous aerosols to the Rocky Mountains. Our findings reveal an important link between wildfire aerosols, air pollution, and remote alpine ecosystems and have important implications for alpine ecosystem health, water quality of high elevation reservoirs, and integrity of snow-fed waters downstream.

## Methods

### Sampling

**Figure 1:**
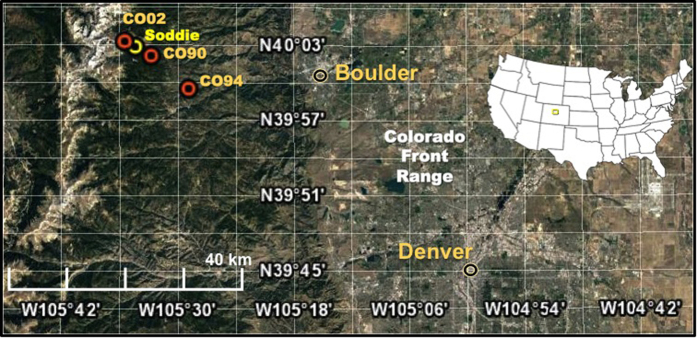
Map of the Niwot Ridge field site, showing the location of the TSP collector at Soddie (NWT-LTER), NADP collection sites at CO02, CO90, and CO94, and Front Range cities of Boulder and Denver, Colorado with map of USA and study site (rectangle) as inset. Both the Soddie and CO02 sites are located in the NWT-LTER. Map data: Google, DigitalGlobe.

**Figure 2:**
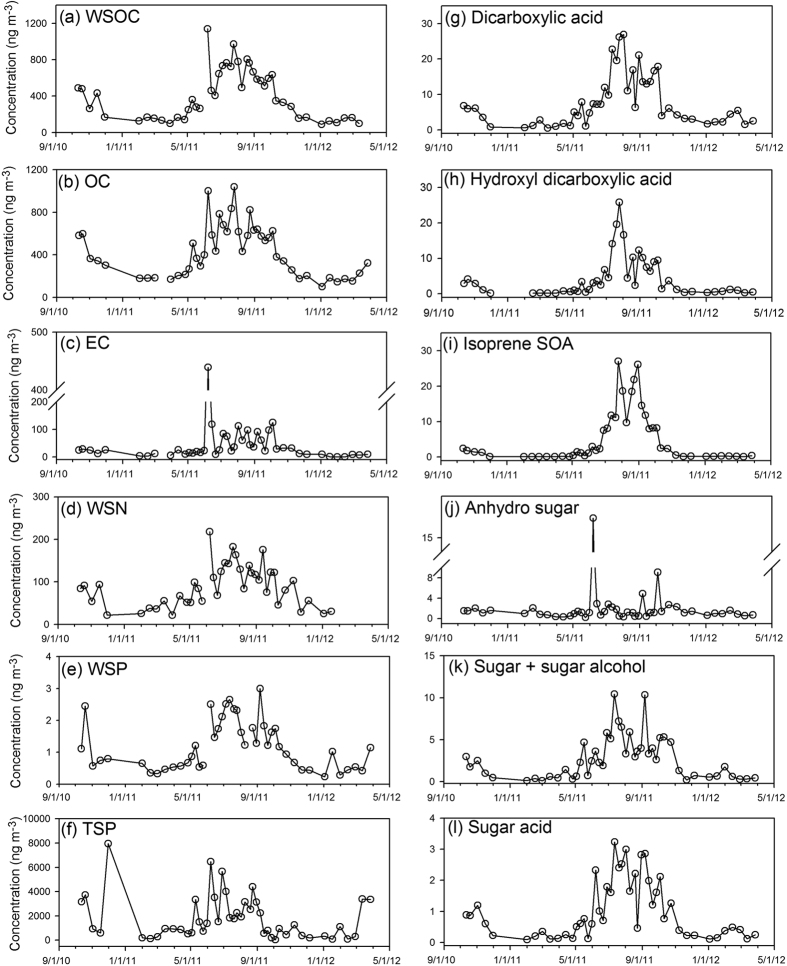
Temporal variations in (**a–f**) bulk components and (**g–l**) groups of WS-OMMs concentrations.

**Figure 3:**
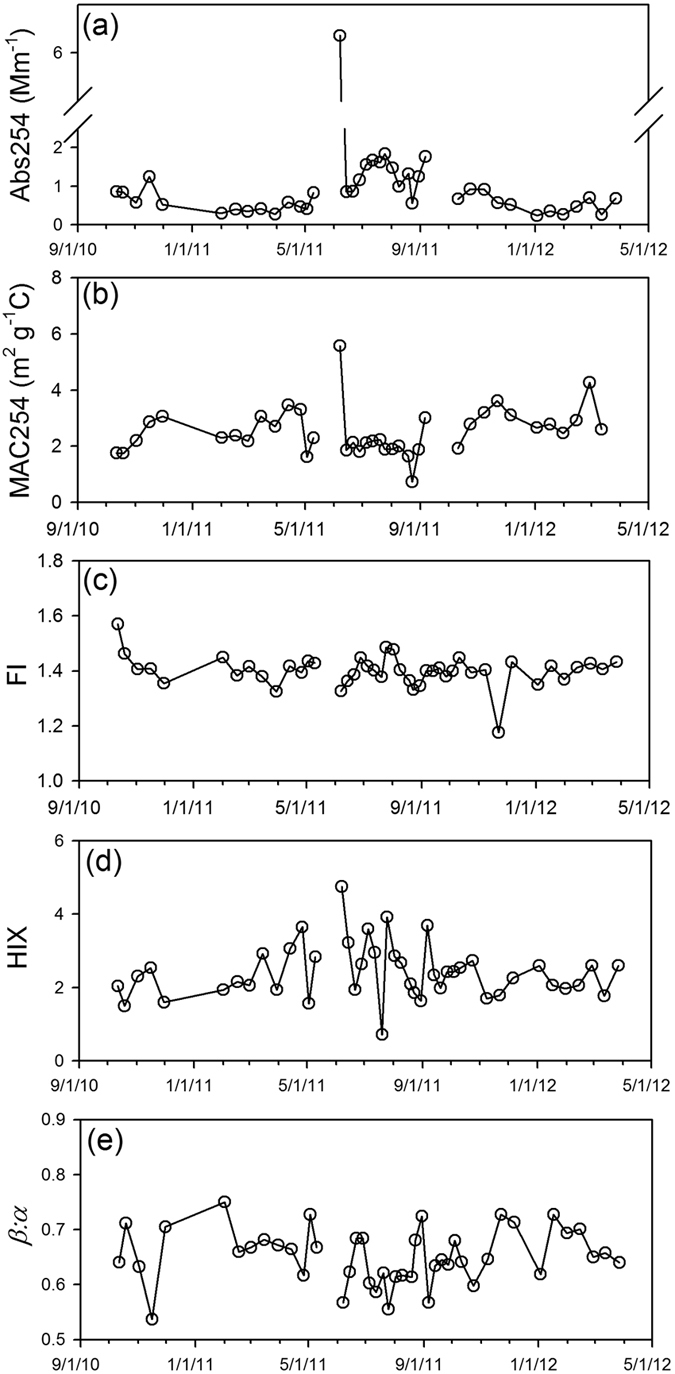
Temporal variations in (**a,b**) UV-light absorbance and (**c–e**) fluorescence characteristics of WSOC.

**Figure 4:**
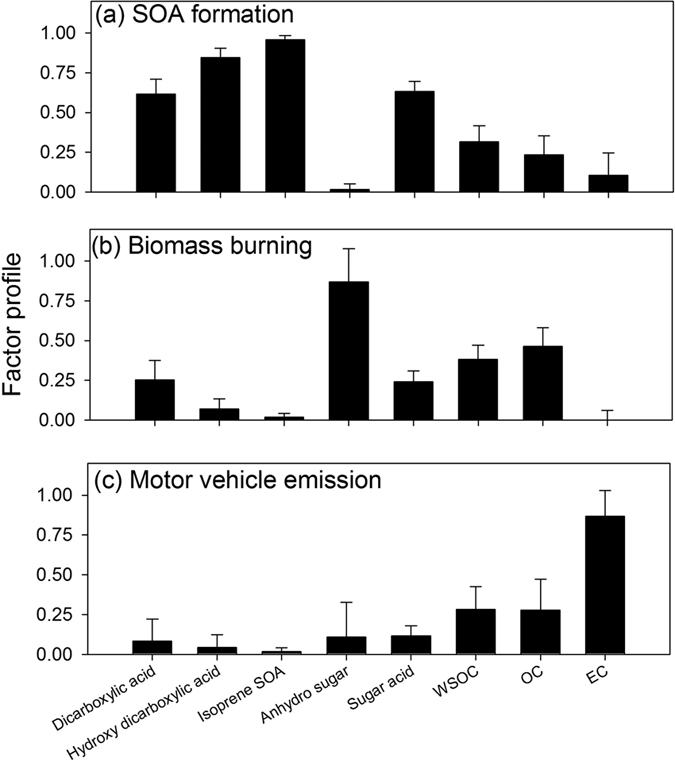
Median PMF factor profiles for the 3-factor solution. The whiskers represent the variability in factor profile derived from bootstrapped PMF solutions (one standard deviation).

**Figure 5:**
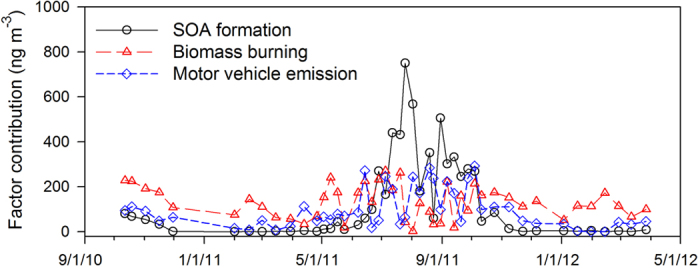
Temporal variations in factor contributions to WSOC derived from the 3-factor PMF solution.

A TSP collector, which is a high volume aerosol collector, was instrumented in late fall 2010 and was co-located at the Soddie atmospheric deposition collection site near tree line at the NWT-LTER (Fig. 1). The Soddie site (40.05° N, 105.57° W) at Niwot Ridge (Fig. 1) is located around 30 km west of Boulder, CO, USA at 3345 m.a.s.l. The TSP collector was equipped with a pre-leached (Millipore deionized water) and pre-combusted (at 450 °C for 6 h) glass fiber filter (0.7 μm nominal pore size, GFF-style) and operated at a flow rate of 1.3–1.4 m^3^ min^−1^ for each sample. Forty-seven filter samples were collected from fall 2010 to spring 2012. Once the samples were collected, they were sealed and returned to the laboratory. The filters were then subsampled for particulate mass determination in a temperature and humidity controlled environment. The TSP mass on a filter was obtained from the difference between pre- and post-sampling filter weights determined on a micro-balance (repeatability, 2 μg). Sampling dates, PM concentrations of each sample, and other sampling details are listed in Supplementary Table S1.

### Water chemistry analysis

A 10 cm^2^ slice of each sample was extracted with 50 mL of Milli-Q water via 30 min shaking at 120 rpm. The water extracts were then filtered through pre-combusted (450 °C for 6 h) 0.70 μm nominal pore size glass fiber filters (Millipore GFF). The instruments and methods used to analyze WSOC, WSN and WSP were provided by Mladenov *et al*.^1^. Briefly, WSOC was measured as non-purgeable organic carbon by high temperature catalytic oxidation on a TOC analyzer (TOC-V CSN, Shimadzu); WSN was also measured using the TOC/TDN analyzer, and WSP was analyzed by persulfate digestion using the Lachat Quick Chem series of instruments. Instrument detection limits were reported in Mladenov *et al*.^1^.

### UV-vis absorbance measurements and transformation

Details of the UV-vis absorbance measurements for water-based solution were given in Mladenov *et al*.^1^. In this work, an aliquot of each sample’s filtered water extract was measured using an Agilent 8453 UV-vis spectrophotometer. UV-vis absorbance spectra between wavelengths of 200 and 900 nm were recorded, and the mean absorbance from 790 to 800 nm was subtracted from all spectral absorbance values to remove scattering effects. The light absorption reported by the UV-vis spectrophotometer can be expressed as:


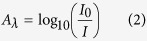


where *A*_*λ*_ is the reported light absorbance at a given wavelength (λ); *I*_*0*_ and *I* are the intensity of the incident and transmitted light, respectively.

The light absorption at a given wavelength (*A*_*λ*_) could be converted to light absorption coefficient (Abs_*λ*_, Mm^−1^) using equation (3):


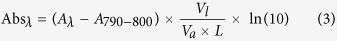


where *A*_790–800_ is the mean absorbance from 790 to 800 nm; *V*_*l*_ (m^3^) is the volume of water (50 mL) used for extraction; *V*_*a*_ (m^3^) is the volume of the sampled air for the extracted slice of filter; *L* (0.01 m) is the optical path length of the quartz cuvette in the UV-vis spectrometer. For ease of analysis, we focus on the absorption at 254 nm as a simple measure of organic matter in collected TSP samples. The bulk mass absorption coefficient of the water extracts at 254 nm (MAC_254_, m^2^ g^−1^C) was calculated as:


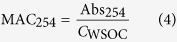


where *C*_WSOC_ is the mass concentration of WSOC (μg m^−3^).

### Fluorescence spectral acquisition

Details on the instruments and method used to acquire fluorescence spectra for water-based solutions were provided by Mladenov *et al*.^1^. In this work, the fluorescent components of TSP water extracts were investigated by measuring their EEMs, which are a 3-dimentional representation of fluorescence intensities scanned over a range of excitation/emission (ex/em) wavelength. The EEM of each sample was collected with a JY-Horiba Spex Fluoromax-3 spectrophotometer at room temperature. All fluorescence spectra were collected in signal-to-reference (S:R) mode with instrumental bias correction. Instrument-specific corrections, Raman area normalization, and Milli-Q blank subtraction were conducted with Matlab (R2009b). The method for the application of PARAFAC modeling to our EEM data set is given in Supplementary Information, and the identified individual fluorescent components loadings are presented in Supplementary Table S2 and Supplementary Fig. S6.

We evaluated changes in fluorescence indices typically applied to aquatic and soil environments in order to further investigate the application of fluorescence spectroscopy to organic aerosols. The two-dimensional fluorescence index (FI), introduced by McKnight *et al*.^65^ to evaluate microbial and terrestrial contributions to DOM in surface water, was calculated as the ratio of emission intensities at 470 nm to 520 nm at an excitation of 370 nm^29^. The humification index (HIX)^70^ is a ratio of the area under the humic-like region (435 to 480 nm), where the carbon:hydrogen (C:H) ratio is high, to the area in the protein-like region (300 to 345 nm) at an excitation of 254 nm. The HIX was developed to estimate the degree of maturation of DOM in soil. The freshness index (*β:α*)^71^ is a ratio of emission intensity at 380 nm to that of the region between 420 and 435 nm at an excitation of 310 nm and was developed to quantify recently produced organic matter in ocean environments. Although none of the widely-used fluorescence indices were originally intended to track sources or transformation of atmospheric aerosols, we determined the FI, HIX and *β:α* for the water extract of each TSP sample to evaluate if there may be relationships present between these indices and sources of organic aerosols.

### OC-EC and WS-OMMs analysis

Details of the bulk OC, EC and WS-OMMs analysis are given in supplementary information. Briefly, the bulk OC and EC on filters were analyzed using a laboratory OC-EC analyzer (Sunset Labs) with the National Institute of Occupational Safety and Health 5040 method^72^. WS-OMMs were extracted from filters using a mixture of methanol and methylene chloride. After filtration, drying and necessary derivatization, the final extract solution was analyzed by GC-MS. Field blanks were collected and analyzed using the identical method. Ignorable background has been observed for OC and EC measurements, and no contamination has been found for WS-OMMs analysis. The concentrations of OC, EC and identified WS-OMMs are provided in Table 1.

### PMF analysis and data selection

The PMF2^73^,^74^ model coupled with a bootstrap technique^75^ was used in this work for source apportionment. This method has been introduced and applied in previous work^43^,^76^. Briefly, PMF uses an uncertainty-weighted least-squares fitting approach to resolve factor profiles and contributions from a series of compositional data of ambient particles. The stationary block bootstrap technique generates 1000 replicate data sets from the original data set and each was analyzed with PMF. Each factor of a bootstrap solution is compared to that of the base case solution by factor profile, so as to generate a rate of factor matching between bootstrapped and base case factors. The factor number was determined by the interpretability of different PMF solutions (3–5 factors) and factor matching rate (at least >50%).

To resolve the sources or processes related to particulate WSOC, bulk OC, EC and the WS-OMMs measured in this work were included for PMF analysis. Due to the small sample number, the WS-OMMs of each class listed in Table 1 were added together as one input species for PMF analysis. The final input species included dicarboxylic acid, hydroxy dicarboxylic acid, isoprene SOA, anhydro sugar, sugar acids, WSOC, OC and EC. The WSN, WSP, sugars and sugar alcohols were not used for PMF analysis due to the implausible PMF solution. To avoid the impact of outliers to PMF analysis, the sample collected during 31 May–7 June 2011 was not included. For that sample, the concentrations of anhydro sugars and EC were unusually high (more than 10 times higher than the average of remains). The uncertainties of chemical species concentrations were estimated by^77^:


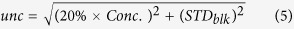


where *Conc.* is the measured concentration of each species and *STD*_*blk*_ is the standard deviation of the field blank measurements. Due to the low field blank contamination, no species concentration is lower than two times of its uncertainty (set as detection limit). The missing species observation was replaced by the geometric mean of the remaining observations, and its accompanying uncertainty was set as four times the geometric mean.

### Air masses transport characteristics

The source of air masses over the Soddie site were also examined by computing backward trajectories (http://ready.arl.noaa.gov/hypub-bin/trajasrc.pl) using the HYSPLIT model (NOAA Air Resources Laboratory) with archived data from the Global Data Assimilation System (GDAS)^78^. Five days of backward trajectory analysis were conducted for the midpoint of each sampling period. GDAS1 was selected as the meteorological data; model vertical velocity was used as the vertical motion calculation method; and the start point was set at 500 m above the ground level. Representative backward trajectory plots for each season were shown in Supplementary Fig. S2.

## Additional Information

**How to cite this article**: Xie, M. *et al*. Water soluble organic aerosols in the Colorado Rocky Mountains, USA: composition, sources and optical properties. *Sci. Rep.* **6**, 39339; doi: 10.1038/srep39339 (2016).

**Publisher's note:** Springer Nature remains neutral with regard to jurisdictional claims in published maps and institutional affiliations.

## Supplementary Material

Supplementary Information

## Figures and Tables

**Table 1:** Measurements of chemical composition (ng m^−3^) of TSP (*N* = 47), UV-light absorbing and fluorescent properties of WSOC.

Species	Median	Mean	Std^a^	Range	% of missed observation^b^
**Bulk components**					
WSOC	348	406	269	88.7–1139	4.26
OC	373	430	240	99.3–1039	2.13
EC	23.3	42.6	68.9	0.0023–439	2.13
WSN	84.1	88.8	48.9	21.3–218	12.8
WSP	1.02	1.18	0.76	0.23–3.00	4.26
TSP	1115	1825	1803	41.2–7947	0.00
**Polar organic tracers**					
*Dicarboxylic acid*					
Succinic acid (C4)^c^	3.32	5.25	5.01	0.23–18.8	0.00
Glutaric acid (C5)^c^	1.10	1.57	1.50	0.12–6.55	0.00
Adipic acid (C6)^c^	0.65	0.99	0.84	0.091–4.15	6.38
*Hydroxy dicarboxylic acid*					
Malic acid^c^	0.91	2.14	2.95	0.063–15.0	6.38
Hydroxyglutaric acid^d^	0.82	1.64	1.96	0.074–7.32	6.38
Hydroxyadipic acid^d^	0.44	0.79	1.02	0.032–4.68	6.38
*Isoprene SOA*					
2-Methylglyceric acid^e^	0.90	1.91	2.64	0.044–12.4	0.00
2-Methylthreitol^e^	0.16	0.89	1.33	0.0061–5.51	2.13
2-Methylerythritol^e^	0.43	2.33	3.57	0.0099–14.3	2.13
*Anhydro sugars*					
Levoglucosan^c^	0.86	1.16	1.69	0.058–10.9	0.00
Mannosan^f^	0.14	0.30	0.46	0.045–2.64	0.00
Galactosane^f^	0.14	0.29	0.49	0.018–2.88	0.00
*Sugars, sugar alcohols and acids*					
Mannose^c^	0.026	0.038	0.046	0.0036–0.21	21.3
Fructose^c^	0.68	1.09	1.02	0.062–4.45	6.38
Glucose^c^	0.29	0.49	0.56	0.017–2.83	12.8
Meso-erythritol^c^	0.049	0.17	0.44	0.0039–2.40	12.8
Arabitol^c^	0.73	1.10	1.06	0.051–4.14	0.00
Glyceric acid^e^	0.52	0.86	0.78	0.082–2.65	0.00
Erythronic acid^e^	0.062	0.13	0.14	0.012–0.56	0.00
Threonic acid^e^	0.034	0.045	0.040	0.0044–0.18	19.1
**UV-light absorbing and fluorescent properties**					
Abs254 (Mm^−1^)	0.69	0.95	0.99	0.24–6.36	14.9
MAC254 (m^2^ g^−1^C)	2.30	2.52	0.84	0.73–5.58	17.0
FI	1.40	1.40	0.057	1.18–1.57	6.38
HIX	2.32	2.42	0.74	0.72–4.75	6.38
*β:α*	0.65	0.65	0.050	0.54–0.75	6.38

^a^standard deviation.

^b^calculated as missed No. of observation/total sample number (*N* = 47) × 100%.

^c^quantified using authentic standards.

^d^quantified using ketopinic acid.

^e^quantified using meso-erythritol.

^f^quantified using levoglucosan.
