# Supplementary material for: Water soluble organic aerosols in the Colorado Rocky Mountains, USA: composition, sources and optical properties
Source: Sci Rep. 2016 Dec 19;6:39339. doi: 10.1038/srep39339 (PMC5171866; doi:10.1038/srep39339)
Supplement: Supplementary Information [file srep39339-s1.pdf]

Supplementary information for:

Water-soluble organic aerosols in the Colorado Rocky Mountains, USA: composition, sources and optical properties

Mingjie Xie[1a], Natalie Mladenov[2]*, Mark W. Williams[3], Jason C. Neff[4], Joseph Wasswa[2], and Michael P. Hannigan[1]

[1]Department of Mechanical Engineering, University of Colorado, Boulder, CO 80309, United States
[2] Department of Civil, Construction, and Environmental Engineering, San Diego State University, San Diego, California 92182, United States
[3]Institute for Arctic and Alpine Research and Department of Geography, University of Colorado, Boulder, CO 80309, United States
[4]Department of Geology, University of Colorado, Boulder, CO 80309, United States

[a] Now at: National Risk Management Research Laboratory, Office of Research and Development, United States Environmental Protection Agency, 109 T.W. Alexander Dr., Research Triangle Park, NC 27709, United States

Correspondence to: Natalie Mladenov
E-mail: nmladenov@sdsu.edu;
Tel: 619-594-0725
Fax: 619-594-6005
Mailing address: 5500 Campanile Drive, San Diego, CA, 92182

Number of pages: 17

Number of tables: 3

Number of figures: 8

Supplementary Methods

1. PARAFAC model application

We applied PARAFAC modeling to further identify the individual fluorescent components in our corrected EEM dataset. PARAFAC modeling of EEMs was conducted in MATLAB using the "drEEM Toolbox" (ver 0.1.0) following the recommendations and procedures of Murphy et al.[1]. To prepare the data for PARAFAC modeling, regions of the spectrum influenced by Rayleigh scatter peaks were removed. After data preparation, outlier identification was performed for 2 to 7 models run with non-negativity constraints. Two samples emerged as outliers and were removed. The model was fit using 3-, 4-, 5- and 6 component models. The 4-, 5- and 6 component models had residual EEMs that contained mostly instrumental noise with minor systematic signals observed in the residual EEMs. The 3-component model had higher peaks in the residual, which result from under-fittting. Random initialization modeling gave core consistency values of 46.4%, 32.9%, 0.99% and -1.3% for a 3, 4, 5 and 6 component models respectively. After further analysis with split validation steps as stated in Murphy et al.[1], only a 4 component model validated. Based on the results from the residual and split validation analysis, we determined that a 4-component model had the best fit. The PARAFAC component loadings are given in Supplementary Table S2.

2. OC-EC analysis.

A 1.5 cm^2 punch taken from each filter sample was loaded on a prebaked punch (1.5 cm^2) of quartz fiber filter and analyzed using the NIOSH method 5040[2,3] on a Sunset Thermal Optical Transmission (TOT) Laboratory ECOC analyzer. The total OC carbon includes

OC1, OC2, OC3, OC4 and PC, representing the carbon measured at four distinct temperature steps (340, 500, 615, and 900 °C) with a pyrolized carbon (PC) adjustment in the first heating cycle of the method. The EC was made up of the carbon measured during the second heating cycle with a final temperature of 910 °C. The OC and EC amounts, total deposition area of the GFF, and sample volume were used to obtain the final concentration. Field blanks were collected and the blank values were more than one order of magnitude lower than the ambient sample with the lowest OC and EC loadings.

3. Analysis of WS-OMMs

Aliquots of each filter were extracted by 20 mL of methanol and methylene chloride mixture (1:1, v/v) ultrasonically two times (15 minutes each). The total extracts were filtered and evaporated to a final volume of ~0.5 mL. After that, the extracts were transferred to a 2 mL glass vial and blown down to dryness under a gentle stream of ultrapure N_2 and reacted with 50 μL of N,O-bis(trimethylsilyl)trifluoroacetamide (BSTFA) containing 1% trimethylchlorosilane (TMCS) and 10 μL of pyridine for 3 h at 70 °C. After cooling down to room temperature, internal standards (hexadecane-d_{34} in hexane) and pure hexane were added before instrumental analysis. The resulting solution was analyzed by an Agilent 6890N gas chromatograph (GC) coupled with an Agilent 5975 mass spectrometer (MS) operated in the electron ionization mode (70 eV). An aliquot of 2 μL of each sample was injected under splitless mode. The GC separation was carried out with a DB-5 ms capillary column (30 m × 0.25 mm × 0.25 μm, Agilent). The GC oven temperature was programmed from 80 °C (hold for 5 min) to 200 °C at 3 °C min^{-1}, and then increased to a final temperature of 300 °C (hold for 10 min) at 15 °C

min^{-1}. Linear calibration curves were derived from five dilutions of quantification standards. Dicarboxylic acids and saccharides were quantified by authentic standards; 2-methyltetrols (2-methylthreitol and 2-methylerythritol) were quantified using meso-erythritol; other SOA tracers (e.g., hydroxyl dicarboxylic acid) were quantified using cis-ketopinic acid (KPA). The species not quantified using authentic standards were identified by the comparison of mass spectra to previously reported data[4-6]. Field blanks were collected and no contamination was observed for identified species. The WS-OMMs quantified in this work were listed in Table 1. Recoveries of those WS-OMMs were obtained by spiking standards onto prebaked filters, followed by extraction and quantification in a same manner as that for ambient samples. Except glutaric acid and adipic acid, other species had average recoveries higher than 70% (70.3 ± 3.87 – 97.9 ± 3.17%). The recoveries of glutaric acid and adipic acid were low (50.1 ± 6.05% and 45.6 ± 4.32%) but stable, and their concentrations were still given. The reported concentrations of WS-OMMs were not adjusted by their recoveries.

4. Absorbance correction

Absorbance spectra were corrected by subtracting the mean of the absorbance from 790 – 800 nm according to Mitchell et al.[7] and Mladenov et al.[8]. Other studies recommend single point absorbance correction using the absorbance at 700 nm[9,10]. We compared the single point absorbance value at 700 nm with the mean for the 790-800 nm range for five random samples in our dataset and found that the difference between the two methods was negligible (< 0.0005 a.u.; Supplementary Table S3).

Supplementary Tables

Table S1. Sampling information and TSP concentration for TSP samples.

Sample ID	Period	Season	Year	Sampling Time (h)	TSP concentration (μg/m^3)
7206019	10/5-10/12/10	fall	2010	168	3.16
7206018	10/12-10/19/10	fall	2010	170	3.73
7206017	10/19-11/2/10	fall	2010	334	0.93
8206011	11/2/-11/16/10	fall	2010	340	0.60
8206013	11/16-11/30/10	fall	2010	355	7.95
8206015	1/18-2/1/11	winter	2011	330	0.19
8206016	2/2-2/16/11	winter	2011	357	0.13
8206014	2/16-3/1/11	winter	2011	306	0.28
8364265	3/1-3/15/11	spring	2011	336	0.95
8364267	3/15-3/30/11	spring	2011	363	0.93
8364266	3/30-4/13/11	spring	2011	334	0.89
8364241	4/13-4/26/11	spring	2011	314	0.52
8364242	4/26-5/3/11	spring	2011	170	0.61
8364243	5/3-5/10/11	spring	2011	161	3.35
8364245	5/10-5/17/11	spring	2011	170	1.50
8364244	5/17-5/24/11	spring	2011	172	0.74
8364246	5/24-5/31/11	spring	2011	162	1.38
8364247	5/31-6/7/11	spring	2011	173	6.47
8364248	6/7-6/14/11	spring	2011	161	3.52
8364232	6/14-6/21/11	summer	2011	168	1.52
8364231	6/21-6/28/11	summer	2011	167	5.66
8364230	6/28-7/5/11	summer	2011	167	4.01
8364229	7/5-7/12/11	summer	2011	168	1.84
8364227	7/12-7/20/11	summer	2011	197	1.76

Table S1. Continued

Sample ID	Period	Season	Year	Sampling Time (h)	TSP concentration (μg/m^3)
8364228	7/20-7/25/11	summer	2011	140	2.25
8364225	7/25-8/2/11	summer	2011	167	1.92
8364226	8/2-8/9/11	summer	2011	173	3.15
8364209	8/9-8/19/11	summer	2011	233	2.54
8364210	8/19-8/23/11	summer	2011	99	4.40
8364213	8/23-8/30/11	summer	2011	171	3.14
8364214	8/30-9/6/11	summer	2011	163	2.25
8364212	9/6-9/13/11	fall	2011	167	0.56
8364211	9/13-9/20/11	fall	2011	169	0.80
8549975	9/20-9/27/11	fall	2011	166	0.20
8549976	9/27-10/4/11	fall	2011	168	0.04
8549977	10/4-10/11/11	fall	2011	168	0.96
8549978	10/11-10/24/11	fall	2011	336	0.44
8549979	10/24/-11/08/11	fall	2011	334	1.26
8549980	11/08-11/22/11	winter	2011	296	0.38
8549981	11/22-12/06/11	winter	2011	338	0.19
8549982	12/06-1/3/12	winter	2011	669	0.34
8549948	1/3-1/17/12	winter	2012	334	0.08
8549949	1/17-1/31/12	winter	2012	341	1.12
8549950	1/31-2/14/12	winter	2012	335	0.09
8549951	2/14-2/28/12	winter	2012	334	0.30
8549952	2/28-3/12/12	winter	2012	335	3.38
8549953	3/12-3/27/12	spring	2012	337	3.35

Table S2. PARAFAC component loadings and distributions for each collection period.

Components:	C1		C2		C3		C4	
Peak locations[a]:	250(300)/412 nm		250/486 nm		270/400 nm		250/400 nm	
Sampling period	loading	%[b]	loading	%[b]	loading	%[b]	loading	%[b]
10/5-10/12/10	0.35	34.2	0.39	37.3	0.14	13.5	0.15	15.0
10/12-10/19/10	0.22	29.3	0.18	23.3	0.22	29.4	0.14	18.1
10/19-11/2/10	0.33	28.7	0.34	29.7	0.28	24.8	0.19	16.8
11/2/-11/16/10	0.22	21.7	0.29	27.8	0.17	16.2	0.35	34.3
11/16-11/30/10	0.39	32.8	0.33	28.2	0.38	32.2	0.080	6.82
1/18-2/1/11	0.36	31.0	0.36	31.1	0.33	28.3	0.11	9.59
2/2-2/16/11	0.35	32.9	0.39	36.1	0.18	16.7	0.15	14.3
2/16-3/1/11	0.35	33.4	0.35	33.5	0.13	13.0	0.21	20.2
3/1-3/15/11	0.33	29.6	0.34	30.2	0.20	18.2	0.25	22.1
3/15-3/30/11	0.26	24.5	0.28	26.6	0.24	22.5	0.28	26.4
3/30-4/13/11	0.34	29.5	0.36	31.3	0.18	16.1	0.26	23.1
4/13-4/26/11	0.30	26.3	0.35	30.3	0.27	23.6	0.23	19.8
4/26-5/3/11	0.32	27.3	0.31	27.2	0.30	26.1	0.22	19.4
5/3-5/10/11	0.26	22.9	0.31	27.4	0.23	20.9	0.32	28.8
5/10-5/17/11	0.28	24.7	0.31	28.0	0.20	17.5	0.33	29.8
5/17-5/24/11	0.19	18.6	0.27	27.2	0.15	15.4	0.39	38.8
5/24-5/31/11[c]	NA	NA	NA	NA	NA	NA	NA	NA
5/31-6/7/11	0.32	29.2	0.39	36.3	0.11	10.4	0.26	24.1
6/7-6/14/11	0.38	35.0	0.37	34.2	0.17	16.0	0.16	14.8
6/14-6/21/11	0.40	34.2	0.37	32.2	0.31	26.7	0.080	6.89
6/21-6/28/11	0.50	46.2	0.37	34.2	0.21	19.8	0.00	0.00
6/28-7/5/11	0.43	41.8	0.38	36.5	0.15	14.8	0.072	6.91
7/5-7/12/11	0.40	36.7	0.39	36.2	0.19	17.7	0.10	9.49
7/12-7/20/11	0.15	14.9	0.21	20.2	0.21	20.2	0.46	44.7
7/20-7/25/11	0.42	38.7	0.44	40.6	0.15	14.0	0.073	6.72
7/25-8/2/11	0.49	45.4	0.39	36.3	0.20	18.7	0.00	0.00
8/2-8/9/11	0.44	43.0	0.34	33.2	0.19	18.7	0.052	5.14
8/9-8/19/11	0.30	27.1	0.37	33.0	0.18	16.1	0.26	23.8
8/19-8/23/11	0.35	34.6	0.29	28.4	0.26	25.3	0.12	11.7
8/23-8/30/11	0.14	16.0	0.18	19.6	0.20	22.1	0.38	42.3
8/30-9/6/11	0.41	37.8	0.35	32.4	0.26	23.4	0.070	6.43
9/6-9/13/11	0.29	27.3	0.32	30.7	0.19	18.3	0.25	23.7
9/13-9/20/11	0.34	31.0	0.32	29.2	0.25	22.6	0.19	17.1
9/20-9/27/11	0.32	29.1	0.36	32.6	0.21	18.9	0.22	19.5
9/27-10/4/11	0.30	26.9	0.32	29.1	0.22	19.5	0.27	24.5
10/4-10/11/11	0.20	18.0	0.27	24.7	0.17	15.7	0.46	41.6
10/11-10/24/11	0.30	27.9	0.33	30.8	0.22	20.6	0.22	20.7
10/24/-11/08/11	0.27	22.1	0.29	24.2	0.38	31.3	0.27	22.4
11/08-11/22/11	0.36	29.9	0.33	28.0	0.33	27.7	0.17	14.3
11/22-12/06/11	0.42	38.9	0.42	38.7	0.16	15.3	0.076	7.07
12/06-1/3/12	0.46	41.7	0.41	37.0	0.22	20.0	0.014	1.28
1/3-1/17/12	0.31	30.5	0.33	32.4	0.15	15.2	0.22	21.9
1/17-1/31/12	0.18	16.6	0.26	24.5	0.20	19.1	0.43	39.8
1/31-2/14/12	0.22	21.8	0.28	26.8	0.25	23.9	0.28	27.5
2/14-2/28/12	0.33	28.8	0.38	33.1	0.24	20.9	0.20	17.2
2/28-3/12/12	0.41	36.9	0.41	37.0	0.21	18.9	0.081	7.23
3/12-3/27/12[c]	NA	NA	NA	NA	NA	NA	NA	NA

[a] Primary peak and secondary peak (in parentheses, if present) excitation/emission wavelength pairs listed for each component; [b] percent of total fluorescent loading; [c] these two samples were outliers in the PARAFAC model.

Table S3. Absorbance values in arbitrary units for single point and mean of 790 – 800 nm wavelengths.

Sample	At 700 nm	Mean of 790-800 nm	Difference
8206015	0.00133	0.00120	0.00013
7206019	0.00300	0.00314	-0.00013
8206013	0.00367	0.00353	0.00015
8364227	0.00608	0.00560	0.00048
8206011	0.00392	0.00368	0.00024

Supplementary Figures

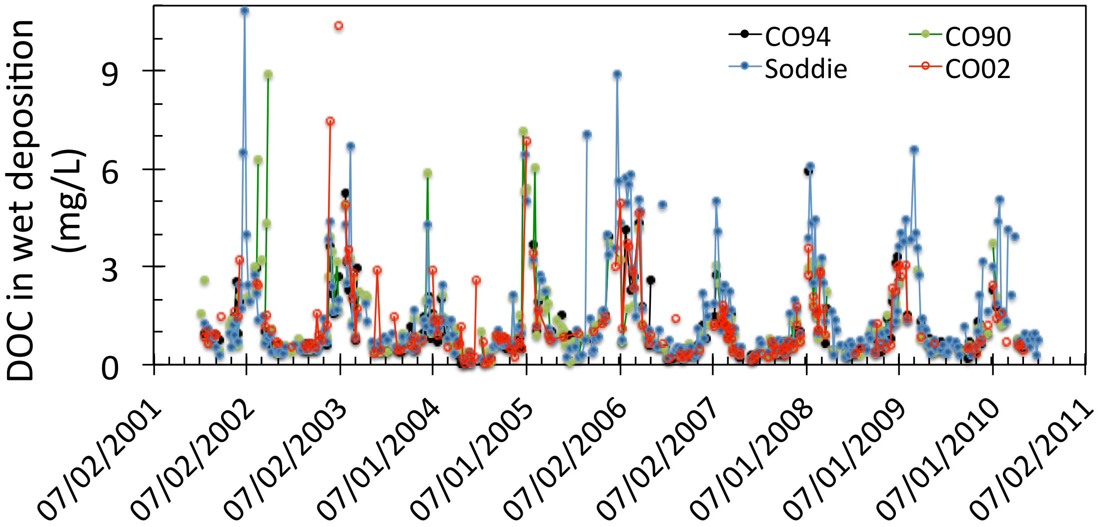

Figure S1. Synchronous patterns in DOC concentrations from 2002 – 2010 in wet deposition at four sites in the Colorado Rocky Mountains: Sugarloaf (CO94), Niwot Ridge-Southeast (CO90), and Niwot Saddle (CO02) NADP sites and the NWT-LTER Soddie site.

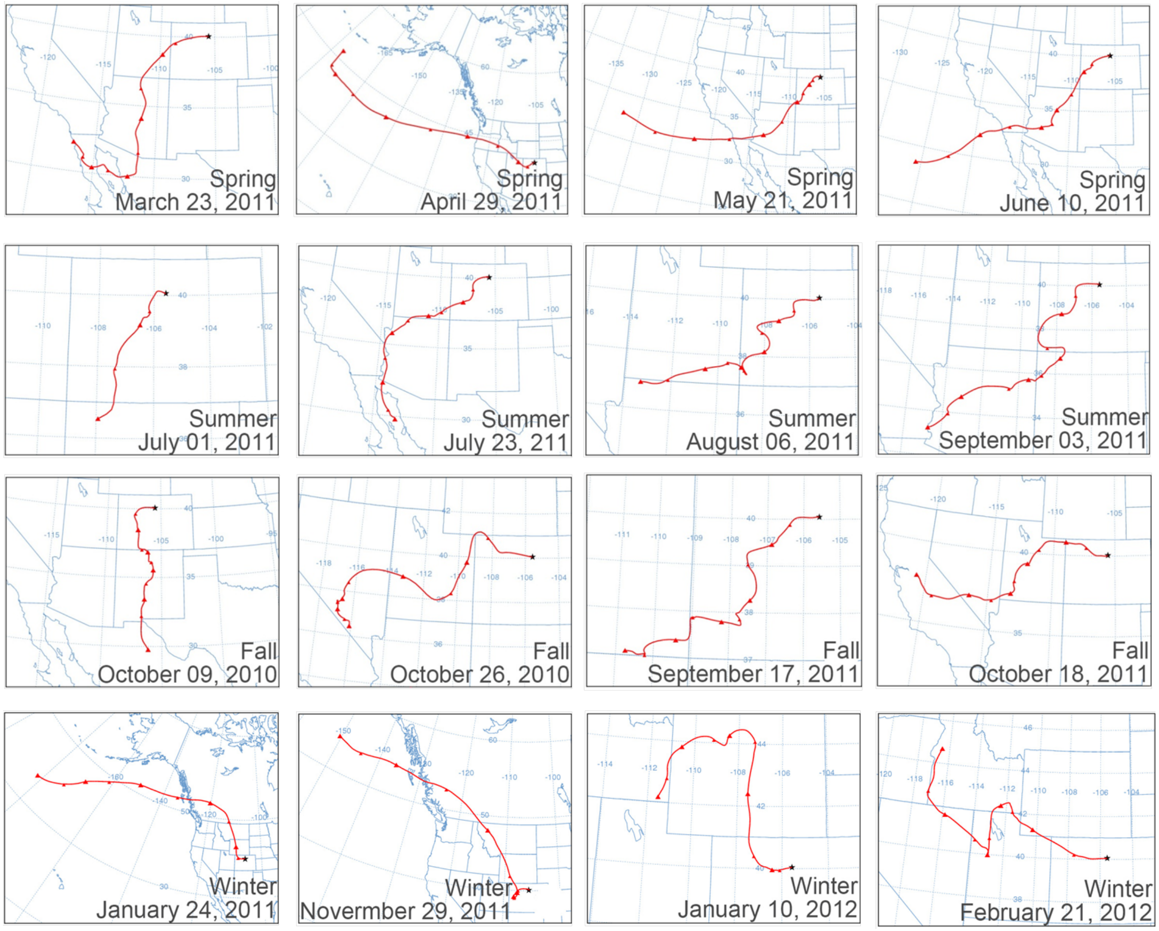

Figure S2. Representative air mass backward trajectories ending on the middle date of selected sampling periods for each season. Each map with backward trajectory plot in this figure was generated using the HYSPLIT model (NOAA Air Resources Laboratory)[11] with archived data from the Global Data Assimilation System (GDAS) on the website (http://ready.arl.noaa.gov/hypub-bin/trajasrc.pl).

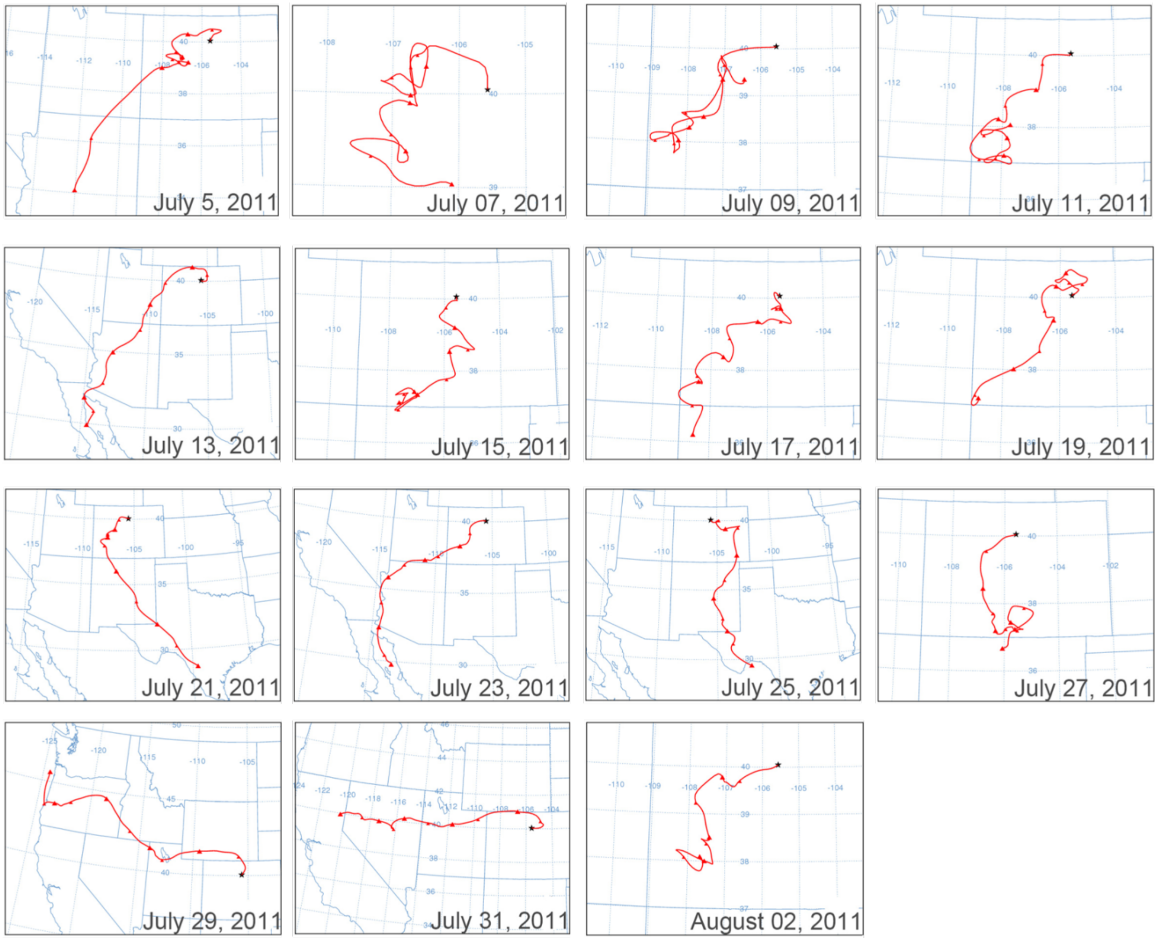

Figure S3. Air mass backward trajectories of ending on every other day from 5 July 2011 to 2 August 2011. Each map with backward trajectory plot in this figure was generated using the HYSPLIT model (NOAA Air Resources Laboratory)[11] with archived data from the Global Data Assimilation System (GDAS) on the website (http://ready.arl.noaa.gov/hypub-bin/trajasrc.pl).

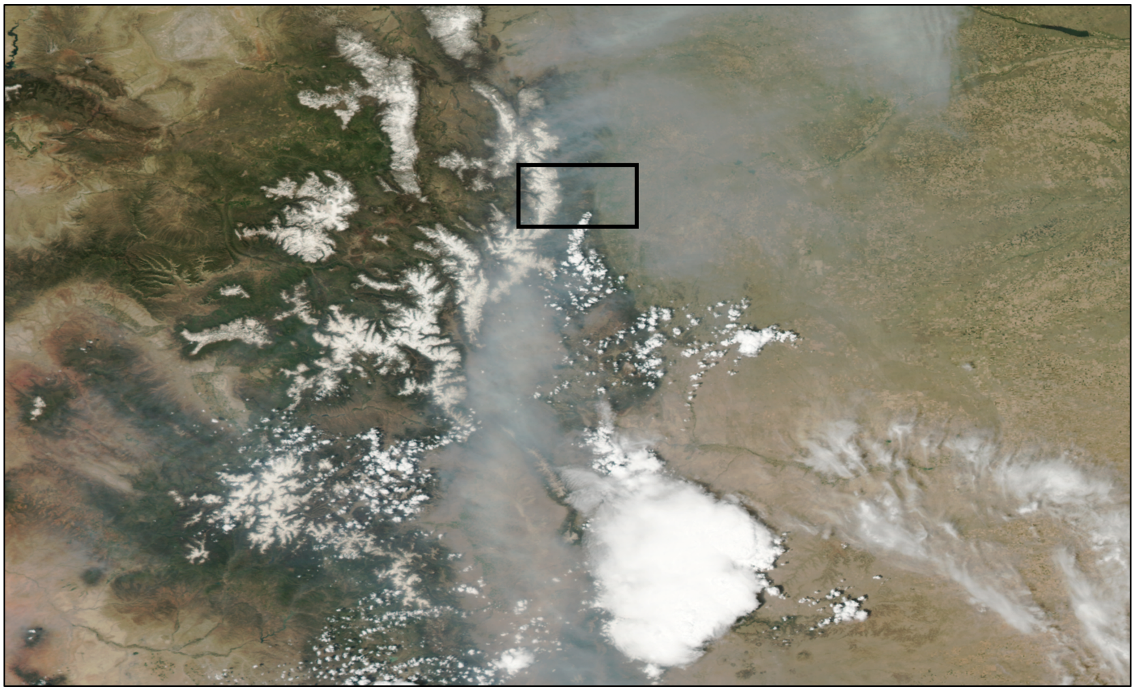

Figure S4. MODIS true color image from the Aqua satellite (http://activefiremaps.fs.fed.us/imagery.php?op=fire&passID=134528) shows smoke plume over Colorado Rocky Mountains on 5 June 2011 at 19:53 GMT. Photo boundaries are boundaries of Colorado state, and rectangle outline denotes same area shown in Figure 1 of the Niwot Ridge study site and adjacent Front Range urban area.

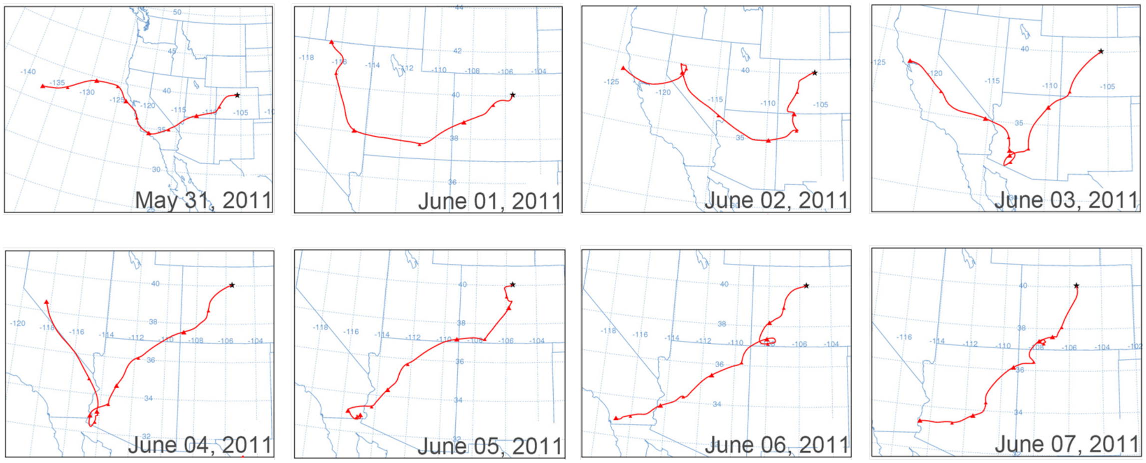

Figure S5. Air mass backward trajectories for each day of the sampling period impacted by wildfire (31 May 2011 to 7 June 2011). Each map with backward trajectory plot in this figure was generated using the HYSPLIT model (NOAA Air Resources Laboratory)[11] with archived data from the Global Data Assimilation System (GDAS) on the website (http://ready.arl.noaa.gov/hypub-bin/trajasrc.pl).

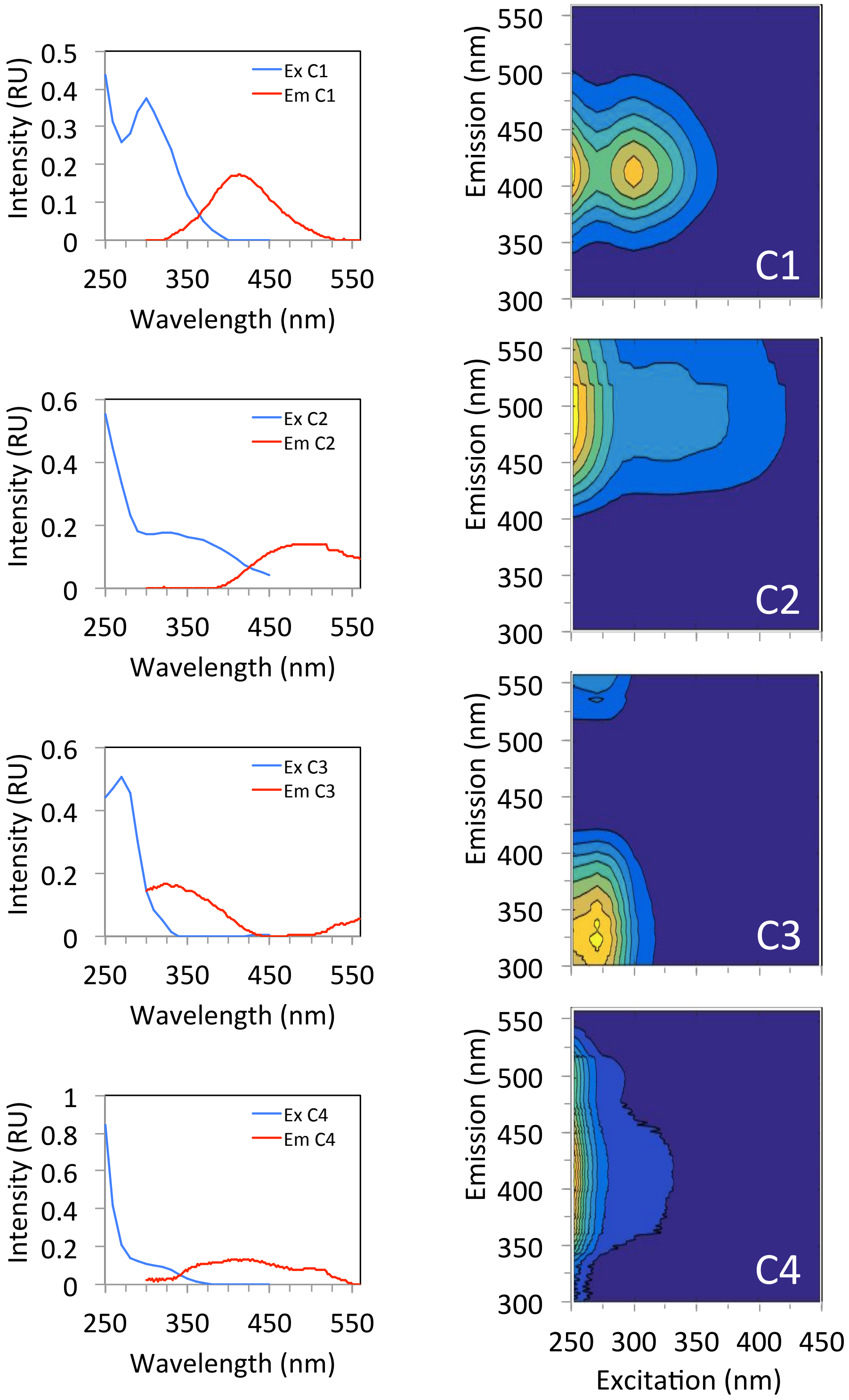

Figure S6. PARAFAC modeling results showing intensities of excitation (blue) and emission (red) of components C1 – C4 (left panel) and EEMs for components C1 – C4 (right panel).

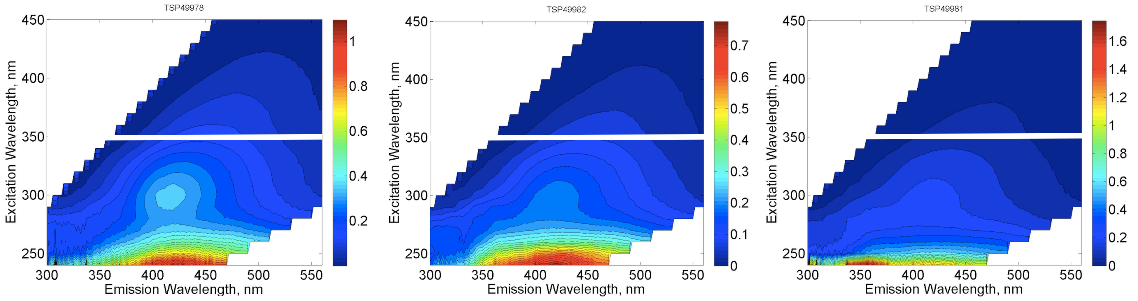

Figure S7. Representative excitation emission matrix (EEM) spectra collected on 24 Oct 2011 (left), 3 Jan 2012 (middle), 6 Dec 2011 (right) show very low fluorescence emission intensities at excitation wavelengths greater than 350 nm (white line).

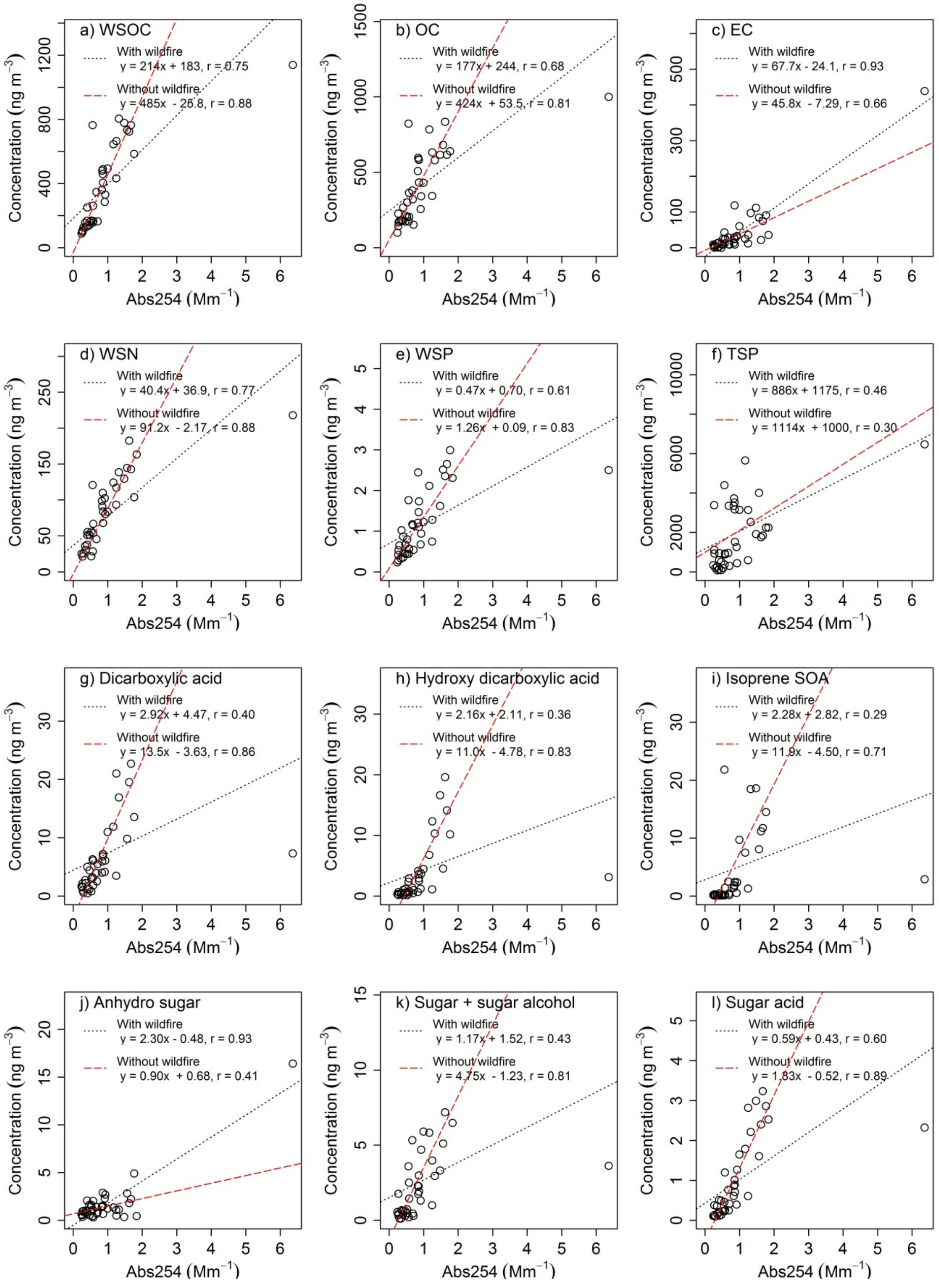

Figure S8. Correlations of (a-f) bulk component concentrations vs. Abs254, and (g-l) concentrations of WS-OMMs vs. Abs254 for datasets with wildfire (black dotted line) and without wildfire (red dashed line) influence.

References

1. Murphy K.R., Stedmon C.A., Graeber D. and R. Bro, Fluorescence spectroscopy and multi-way techniques. PARAFAC, *Anal. Methods*, **5**, 6557-6566, DOI:10.1039/c3ay41160e (2013).
2. NIOSH. Method 5040, Issue 3: Diesel Particulate Matter (as Elemental Carbon). *NIOSH Manual of Analytical Methods (NMAM)*, 4th, ed.; National Institute of Occupational Safety and Health: Cincinnati, OH, (2003).
3. Schauer, J. J. *et al.* ACE-Asia intercomparison of a thermal-optical method for the determination of particle-phase organic and elemental carbon. *Environ. Sci. Technol.* **37**, 993-1001, doi:10.1021/es020622f (2003).
4. Claeys, M. *et al.* Formation of secondary organic aerosols through photooxidation of isoprene. *Science* **303**, 1173-1176, doi:10.1126/science.1092805 (2004).
5. Claeys, M. *et al.* Hydroxydicarboxylic acids: Markers for secondary organic aerosol from the photooxidation of α-pinene. *Environ. Sci. Technol.* **41**, 1628-1634, doi:10.1021/es0620181 (2007).
6. Kleindienst, T. E. *et al.* Estimates of the contributions of biogenic and anthropogenic hydrocarbons to secondary organic aerosol at a southeastern US location. *Atmos. Environ.* **41**, 8288-8300, doi:10.1016/j.atmosenv.2007.06.045 (2007).
7. Mitchell, B.G., *et al*. Ocean Optics Protocols for Satellite Ocean Color Sensor Validation, Revision 4, Volume IV: Inherent Optical Properties: Instruments, Characterizations, Field Measurements and Data Analysis Protocols. J.L. Muellel; G.S. Fargion, and C.R. McClain, Editors, NASA Report. NASA/TM-2003-211621/Rev4-Vol.IV. (2003).
8. Mladenov, N., *et al.* Dust inputs and bacteria influence dissolved organic matter in clear alpine lakes. *Nature Communications*. **2:405** , DOI: 10.1038/ncomms1411, (2011).
9. Srinivas, B. B., and M. M. Sarin,, Light absorbing organic aerosols (brown carbon) over the tropical Indian Ocean: impact of biomass burning emissions *Environ. Res. Lett*. **8**, 044042 (2013).
10. Hecobian, A., *et al.* Water-Soluble Organic Aerosol material and the light-absorption characteristics of aqueous extracts measured over the Southeastern United States. *Atmos. Chem. Phys*., **10**, 5965–5977 (2010).
11. Stein, A.F., Draxler, R.R, Rolph, G.D., Stunder, B.J.B., Cohen, M.D. & Ngan, F. NOAA's HYSPLIT atmospheric transport and dispersion modeling system, *Bull. Amer. Meteor. Soc*. **96**, 2059-2077, http://dx.doi.org/10.1175/BAMS-D-14-00110.1 (2015).
